# Maternal Dppa2 and Dppa4 are dispensable for zygotic genome activation but important for offspring survival

**DOI:** 10.1242/dev.200191

**Published:** 2021-12-21

**Authors:** Oana Kubinyecz, Fatima Santos, Deborah Drage, Wolf Reik, Melanie A. Eckersley-Maslin

**Affiliations:** 1 Epigenetics Programme, Babraham Institute, Cambridge CB22 3AT, UK; 2 Wellcome Trust Sanger Institute, Hinxton CB10 1SA, UK; 3 Centre for Trophoblast Research, University of Cambridge, Cambridge CB2 3EG, UK; 4 Peter MacCallum Cancer Centre, Melbourne, Victoria 3000, Australia; 5 Sir Peter MacCallum Department of Oncology, The University of Melbourne, Victoria 3010, Australia; 6 Department of Anatomy and Physiology, University of Melbourne, Victoria 3010, Australia

**Keywords:** Dppa2, Dppa4, Embryo, Epigenetics, Maternal factors, Zygotic genome activation, Mouse

## Abstract

Zygotic genome activation (ZGA) represents the initiation of transcription following fertilisation. Despite its importance, we know little of the molecular events that initiate mammalian ZGA *in vivo*. Recent *in vitro* studies in mouse embryonic stem cells have revealed developmental pluripotency associated 2 and 4 (Dppa2/4) as key regulators of ZGA-associated transcription. However, their roles in initiating ZGA *in vivo* remain unexplored. We reveal that Dppa2/4 proteins are present in the nucleus at all stages of preimplantation development and associate with mitotic chromatin. We generated conditional single and double maternal knockout mouse models to deplete maternal stores of Dppa2/4. Importantly, *Dppa2/4* maternal knockout mice were fertile when mated with wild-type males. Immunofluorescence and transcriptome analyses of two-cell embryos revealed that, although ZGA took place, there were subtle defects in embryos that lacked maternal Dppa2/4. Strikingly, heterozygous offspring that inherited the null allele maternally had higher preweaning lethality than those that inherited the null allele paternally. Together, our results show that although Dppa2/4 are dispensable for ZGA transcription, maternal stores have an important role in offspring survival, potentially via epigenetic priming of developmental genes.

## Abstract (precis)

**Summary:** Conditional knockout mouse models reveal maternal stores of Dppa2 and Dppa4 are dispensable for zygotic genome activation, contrasting findings in embryonic stem cells. However, both maternal and zygotic Dppa2/4 are required for development.

## INTRODUCTION

One of the first developmental milestones following fertilisation is transcriptional activation of the embryonic genome. This process, termed zygotic genome activation (ZGA) is part of the maternal-to-zygotic transition and considered essential for development to progress. It occurs in two phases: a minor wave at the late zygote/early two-cell stage and a major wave at the middle to late two-cell stage in mouse (four- to eight-cell stage in humans). At the time of ZGA, the preimplantation embryo undergoes dramatic epigenetic reprogramming (reviewed by Eckersley-Maslin et al., 2018); however, we have limited understanding of the maternal factors that regulate ZGA and epigenetic reprogramming *in vivo*, mostly due to complex genetics and limited cell numbers.

More recently, the field has modelled ZGA using a spontaneously occurring rare subpopulation of mouse embryonic stem cells (ESCs), termed 2C-like cells (Macfarlan et al., 2012). These cells share many of the epigenetic and transcriptional features of the two-cell embryo (reviewed by Eckersley-Maslin et al., 2018; Genet and Torres-Padilla, 2020) and recent studies have exploited these similarities to screen for regulators of ZGA-associated transcription *in vitro* (Alda-Catalinas et al., 2020; Eckersley-Maslin et al., 2019; Grow et al., 2021; Hu et al., 2020; Rodriguez-Terrones et al., 2018; Zhao et al., 2018). From these and other studies, the dimerising nuclear proteins developmental pluripotency associated 2 (Dppa2) and 4 (Dppa4) were identified as potent inducers of the 2C-like state (De Iaco et al., 2019; Eckersley-Maslin et al., 2019; Yan et al., 2019). In ESCs, Dppa2/4 directly bind to and induce transcription of the *Dux* transcription factor gene, which in turn regulates expression of downstream ZGA targets (De Iaco et al., 2017; Hendrickson et al., 2017; Whiddon et al., 2017). In addition, Dppa2/4 also regulate other transcripts outside of the Dux network, including LINE-1 elements (De Iaco et al., 2019; Gretarsson and Hackett, 2020) and a subset of bivalently marked developmental genes (Eckersley-Maslin et al., 2020; Gretarsson and Hackett, 2020).

*Dppa2/4* transcripts are present in mature oocytes, further upregulated during ZGA and persist throughout preimplantation development, only for their transcription to be silenced by DNA methylation upon gastrulation at embryonic day (E)6.5 (Eckersley-Maslin et al., 2019; Maldonado-Saldivia et al., 2007). Although single and double *Dppa2/4* zygotic knockout mice survive embryogenesis, they succumb to lung and skeletal defects shortly after birth, despite the gene products not being expressed in these tissues (Madan et al., 2009; Nakamura et al., 2011). This suggests they act by priming the epigenetic landscape earlier in development to enable successful development to take place (reviewed by Eckersley-Maslin, 2020; Watabe, 2012; Zlotorynski, 2020). However, the role Dppa2/4 have in preimplantation embryos remains elusive as current zygotic knockout models are confounded by maternal deposits of these proteins, and homozygous *Dppa2/4* knockout mice have near 100% lethality.

To assess the importance of maternal Dppa2/4 in early embryonic development, including ZGA, we generated conditional single and double knockout mouse models to deplete maternal stores of Dppa2/4 in growing oocytes. Maternal knockout *Dppa2/4* mice are fertile and give rise to viable offspring. Furthermore, molecular analyses reveal that ZGA takes place in two-cell embryos derived from maternal knockouts, indicating that Dppa2/4 is not required to initiate ZGA *in vivo*. However, heterozygote offspring from maternal knockouts have increased preweaning mortality compared with those from paternal knockouts. Therefore, although dispensable for ZGA to take place, maternal stores of Dppa2/4 have an important role in offspring survival.

## RESULTS

### Dppa2/4 proteins localise to euchromatin in preimplantation embryos and associate with mitotic chromatin

**Fig. 1.**
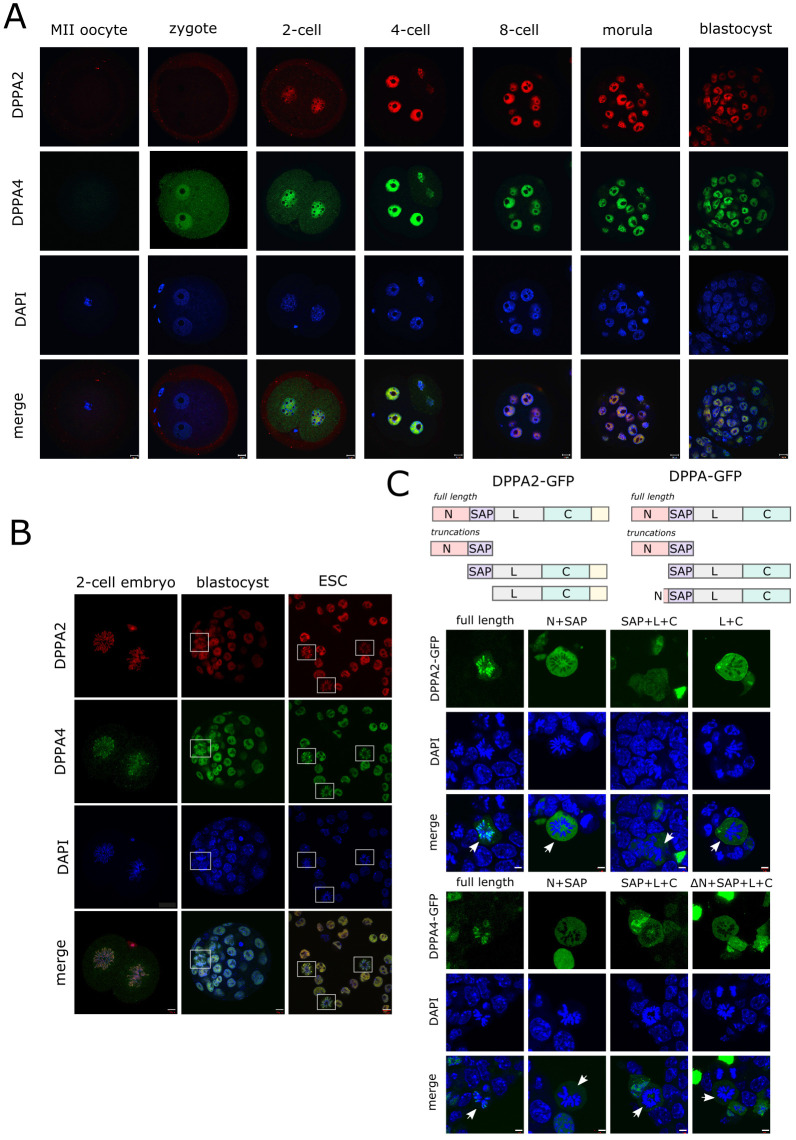
**Dppa2/4 localise to euchromatin in preimplantation embryos and associate with mitotic chromatin.** (A) Immunofluorescence staining of WT mouse MII oocytes and preimplantation embryos with Dppa2 (red), Dppa4 (green) and DAPI nuclear stain (blue). (B) Immunofluorescence staining of WT two-cell embryos (left), blastocysts (middle) or mouse ESCs (right) for Dppa2 (red), Dppa4 (green), DAPI nuclear stain (blue). Mitotic cells are denoted by boxes. (C) Top: schematic of truncations analysed (not to scale). N-terminus (N), SAP, linker (L) or C-terminus (C) domains are denoted. Middle/bottom: localisation of Dppa2-GFP (middle) or Dppa4-GFP (bottom) constructs in mouse ESCs. Full length (left) or truncations (right three) were transiently transfected into cells. Arrows denote mitotic cells. Scale bars: 10 µm (A,B); 5 µm (C).

*Dppa2/4* transcripts are present in growing and mature oocytes and throughout preimplantation development (Eckersley-Maslin et al., 2019) (Fig. S1A); however, protein localisation has not been systematically assessed at these developmental stages. Immunofluorescence staining on wild-type (WT) C57Bl/6 mouse embryos derived from natural matings confirmed the presence of Dppa4, but not Dppa2, in MII oocytes and zygotes (Fig. 1A). Dppa4 protein was present in both pronuclei by postnatal day (P)3 before the minor wave of ZGA (Fig. S1B). There was a marked increase in protein levels from the two-cell stage onwards following DNA replication (Fig. S1C) consistent with the transcriptional upregulation of Dppa2/4 during the major wave of ZGA (Fig. S1A). Protein levels and nuclear localisation of Dppa2/4 remained constant and in all blastomeres from the two-cell stage through to the morula and blastocyst stage. At all stages, there was colocalisation of Dppa2 and Dppa4 proteins with each other consistent with them forming heterodimers (Nakamura et al., 2011).

In interphase cells, Dppa2/4 protein was predominantly euchromatic, as previously reported (Masaki et al., 2007, 2010; Nakamura et al., 2011). Remarkably, we observed strong Dppa2/4 binding to mitotic chromatin in both embryos and ESCs (Fig. 1B), consistent with recent proteomic profiling of mitotic chromatin in ESCs (Djeghloul et al., 2020). To determine whether there was a particular region of Dppa2/4 that was responsible for this mitotic binding, we analysed a series of GFP-tagged truncated constructs in ESCs (Fig. 1C). Dppa2/4 contain a SAP domain and a C-terminal domain that bind RNA/DNA and histones, respectively (Masaki et al., 2010). Although full length Dppa2 and Dppa4 bound mitotic chromatin, all truncations analysed lost this ability despite retaining their nuclear localisation in non-mitotic cells. This indicates that multiple regions of the protein, including the SAP domain, N-terminus and C-terminus, are required together for mitotic binding, and that neither domain on its own is sufficient.

### Maternal *Dppa2/4* single and double knockout mice are fertile

**Fig. 2.**
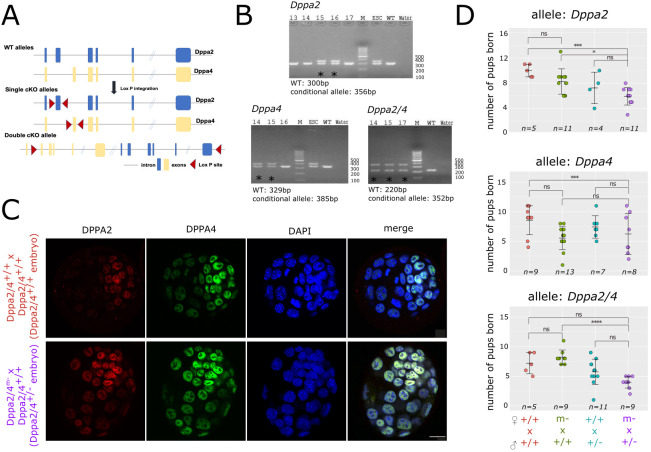
**Maternal Dppa2/4 single and double knockout mice are fertile.** (A) Schematic of conditional knockout alleles (cKO, not to scale) showing exons (boxes) bounded by LoxP sites (red triangles), *Dppa2* allele is shown in blue, *Dppa4* allele in yellow. (B) Genotyping PCR confirming successful generation of heterozygous mice for *Dppa2* (top), *Dppa4* (bottom left) or *Dppa2/4* (bottom right) conditional allele. Asterisk denotes heterozygous founding animals. (C) Immunofluorescence staining of blastocysts collected from Dppa2/4^+/+^ (top) or Dppa2/4^m−^ (bottom) females crossed with Dppa2/4^+/+^ fathers for Dppa2 (red), Dppa4 (green) and DAPI nuclear stain (blue). Additional embryos are in Fig. S2A. (D) Litter size for *Dppa2* (top), *Dppa4* (middle) or *Dppa2/4* (bottom) conditional knockout alleles. Each dot represents an individual litter. Error bars represent mean±s.d. Number of litters analysed (*n*) is shown. *P<0.05, ***P<0.001, ****P<0.0001 (one-way Anova with Tukey multiple comparisons test). ns, non significant. Scale bar: 20 µm.

Having validated the presence of Dppa2/4 protein in preimplantation embryos, we next generated conditional knockout mouse models for *Dppa2/4*. For the single conditional knockouts, LoxP sites were introduced either side of exon 2 of *Dppa2* or *Dppa4* (Fig. 2A; supplemental Materials and Methods). *Dppa2/4* genes are located in tandem on mouse chromosome 16 with no annotated intervening genes. This allowed us to generate a conditional double knockout mouse model by introducing LoxP sites upstream of *Dppa4* exon 2 and downstream of *Dppa2* exon 7 (Fig. 2A). Conditional knockout mice (Fig. 2B) were crossed with Zp3-Cre males to delete *Dppa2* and/or *Dppa4* in growing oocytes and generate maternal knockouts (denoted as Dppa2^m−^ and Dppa4^m−^, and Dppa2/4^m−^ for the single and double maternal knockouts, respectively).

We first assessed whether maternal stores of Dppa2/4 are required to form blastocysts. We crossed control or Dppa2/4^m−^ females with Dppa2/4^+/+^ males and collected the heterozygous Dppa2/4^+/−^ embryos at E3.5. We readily collected phenotypically normal blastocysts from Dppa2/4^m−^ females (Fig. 2C; Fig. S2A). These embryos expressed Dppa2/4 protein from the paternally inherited WT allele, suggesting that ZGA had taken place. Therefore, maternal Dppa2/4 is not required for blastocyst development.

To determine whether maternal Dppa2/4 are required for complete embryonic development, we crossed the Dppa2^m−^, Dppa4^m−^ and Dppa2/4^m−^ with WT males and monitored litter size and survival. All three genotypes repeatedly gave rise to live litters when crossed with WT males (Fig. 2D). There was a tendency for the resulting litter sizes to be smaller, suggesting decreased offspring survival; however, additional matings need to be analysed to assess this comprehensively. In summary, these results indicate that maternal stores of Dppa2/4 are not required to generate live offspring.

### Two-cell embryos from maternal knockout females undergo successful ZGA with subtle differences

**Fig. 3.**
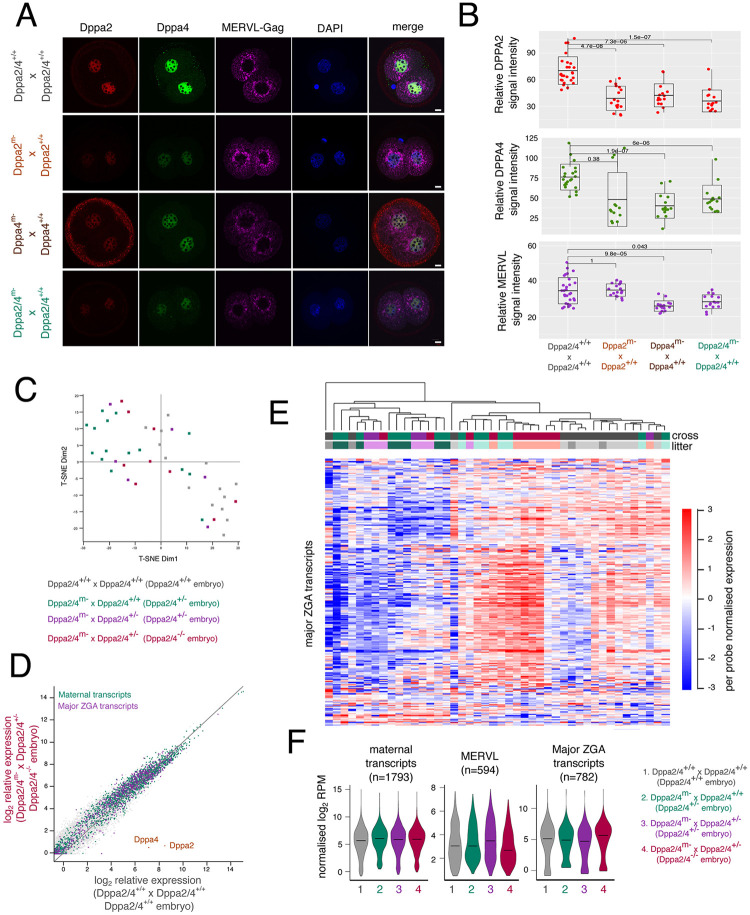
**Two-cell embryos from maternal knockout females undergo successful ZGA with subtle differences.** (A) Immunofluorescence staining of two-cell embryos derived from Dppa2/4^+/+^, Dppa2^m−^, Dppa4^m−^ or Dppa2/4^m−^ females crossed with WT fathers, for Dppa2 (red), Dppa4 (green), MERVL-GAG protein (magenta) and DAPI nuclear stain (blue). Single *z*-slices shown. (B) Relative quantification of MERVL cytoplasmic signal intensity of two-cell embryos in A. Differences are statistically significant and *P*-values denoted (Wilcoxon test with Bonferroni correction for multiple comparisons). (C) T-SNE plot of individual two-cell embryos that are Dppa2/4^+/+^ (grey), Dppa2/4^+/−^ (green, purple) or Dppa2/4^−/−^ (red). (D) Scatterplot between Dppa2/4^+/+^ and Dppa2/4^−/−^ two-cell embryos highlighting maternal transcripts (green) and major ZGA transcripts (purple). (E) Per probe normalised heatmap showing relative expression of major ZGA transcripts in two-cell embryos. Genotype and litter of the embryos are denoted and do not cluster together. (F) Normalised expression of maternal transcripts, MERVL and major ZGA transcripts in the different embryo genotypes. Violin plot shows kernel density plot with median shown (black line). Scale bars: 10 µm.

Although Dppa2/4^m−^ females give rise to offspring, their litters had a tendency to be smaller than their WT counterparts. Therefore, we analysed two-cell embryos at a molecular level to determine whether there were any ZGA defects. First, we collected two-cell embryos from WT or maternal knockout females of all three genotypes crossed with WT males and performed immunofluorescence for the MERVL endogenous retrovirus GAG protein (Fig. 3A) which is used as a marker for ZGA (Kigami et al., 2003; Peaston et al., 2004). As expected, embryos from Dppa2/4^+/+^×Dppa2/4^+/+^ crosses had high levels of cytoplasmic MERVL GAG protein, indicating that ZGA had taken place. Heterozygous Dppa2^+/−^, Dppa4^+/−^ and Dppa2/4^+/−^ embryos from maternal knockout females all had reduced yet detectable levels of Dppa2 and Dppa4 proteins (Fig. 3A,B), indicating expression from the paternal allele. Furthermore, in the single knockouts the absence of one protein led to destabilisation of the other, consistent with what has been observed in ESCs (Eckersley-Maslin et al., 2019; Eckersley-Maslin et al., 2020). Notably, all embryos derived from maternal knockout females crossed with WT males had detectable MERVL GAG protein, indicating ZGA had successfully taken place. However, both single Dppa4^+/−^ and double Dppa2/4^+/−^ embryos had lower MERVL levels than their Dppa2/4^+/+^ counterparts (Fig. 3A,B), suggesting that DPPA4 may have a subtle role in modulating ZGA.

To determine more globally whether there are any defects in ZGA, we performed single-embryo RNA-seq to comprehensively survey the transcriptome in two-cell embryos. Three matings were set up: Dppa2/4^+/+^ females with Dppa2/4^+/+^ males as controls in which all embryos would be Dppa2/4^+/+^; Dppa2/4^m−^ females with Dppa2/4^+/+^ males in which all embryos would be Dppa2/4^+/−^; and Dppa2/4^m−^ females with Dppa2/4^+/−^ males in which half the embryos would be expected to be Dppa2/4^+/−^ and half Dppa2/4^−/−^. We analysed levels of *Dppa2* and *Dppa4* transcripts to assign embryos from the latter category into the two embryo genotypes so they could be analysed separately (Fig. S3A). Clustering analysis using t-SNE revealed that two-cell embryos derived from Dppa2/4^m−^ females had a very similar transcriptome to those from Dppa2/4^+/+^ females (Fig. 3C). Consistently, there were just 93 differentially expressed genes between Dppa2/4^+/+^ and Dppa2/4^−/−^ embryos, and only six genes including *Dppa4* that were consistently differentially expressed across all heterozygous and homozygous embryos (Fig. S3B-D; Table S1). Significantly, there were no substantial differences in the expression of major ZGA transcripts between Dppa2/4^−/−^ and Dppa2/4^+/+^ embryos, indicating that ZGA had successfully taken place (Fig. 3D-F; Fig. S3F; Table S1). Moreover, maternally deposited transcripts remained unchanged (Fig. 3F). In contrast to MERVL protein levels (Fig. 3A,B), *MERVL* transcription was unaltered (Fig. 3F; Table S1), suggesting there may be differences in protein translation and/or stability in Dppa2/4^+/−^ embryos which warrants further investigation in future studies. Alternatively, Dppa2/4 may have more subtle effects in ZGA reflected by the immunofluorescence staining but not captured by the transcriptome analysis. Lastly, we compared our *in vivo* results with the *in vitro* 2C-like cell system and found no consistent differences in *Dux*, nor 2C-like genes in the knockout embryos compared with control (Fig. S3F,G). Together, our results reveal that embryos from maternal knockout embryos undergo successful ZGA and were largely transcriptionally indistinguishable from those from WT females. Therefore, neither maternal nor zygotic Dppa2/4 is essential for ZGA *in vivo*.

### Both maternal and zygotic Dppa4 are required for offspring survival

**Fig. 4.**
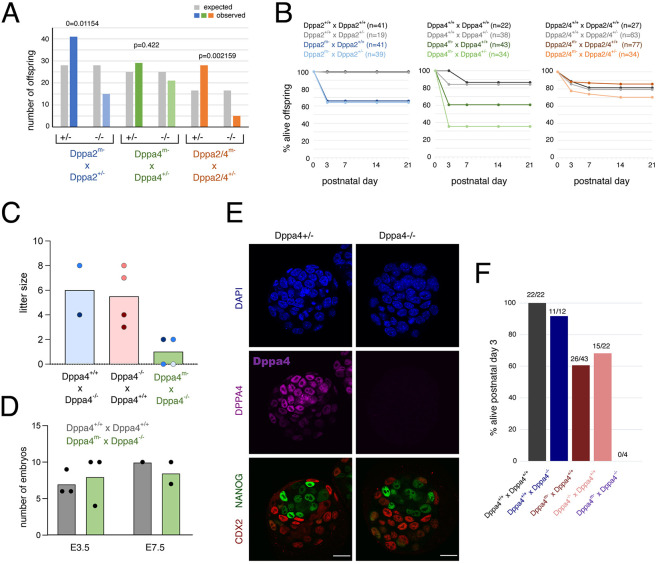
**Both maternal and zygotic Dppa4 are required for offspring survival.** (A) Genotype frequency of offspring (dead and alive) from Dppa2^m−^ (blue, *n*=56 offspring from 11 matings), Dppa4^m−^ (green, *n*=50 offspring from 12 matings) or Dppa2/4^m−^ (orange, *n*=36 offspring from nine matings) females crossed with the respective heterozygous males. Grey bars represent expected Mendelian ratios. Dppa2 and Dppa2/4 difference is significant (Chi-Square test). (B) Percentage of live offspring at P0, P3, P14 and P21 for WT females (grey) and maternal knockout females (coloured) crossed with WT males (dark) or heterozygous males (light) for *Dppa2* (blue), *Dppa4* (green) or *Dppa2/4* (orange) alleles. Number of live pups at P0 is denoted for each cross. (C) Litter size (dead and alive) for Dppa4^−/−^ males (blue) or Dppa4^−/−^ females (pink) mated with Dppa4^+/+^ animals. Also shown are Dppa4^m−^ females crossed with Dppa4^−/−^ males (green). The same animals were used in several matings and are denoted using light/medium/dark blue (for males) or pink (for females). (D) Number of embryos collected at E3.5 or E7.5 from Dppa4^+/+^ females crossed with Dppa4^+/+^ males (grey, embryos are Dppa4^+/+^) or Dppa4^m−^ females crossed with Dppa4^−/−^ males (green, embryos are Dppa4^−/−^). (E) Immunofluorescence staining of Dppa4^+/+^ or Dppa4^−/−^ embryos collected at E3.5 for Dppa4 (magenta), CDX2 (red) and NANOG (green) counterstained with DAPI nuclear stain (blue). (F) Proportion of offspring alive at P3 for control (grey) and heterozygous Dppa4^+/−^ offspring when the null allele is inherited paternally (blue) or maternally (red, pink). No live offspring were born from crosses between Dppa4^m−^ and Dppa4^−/−^ animals (purple). Fraction above each bar represents total live offspring at P3 over total number of offspring born. Scale bars: 20 µm.

Despite undergoing successful ZGA, maternal Dppa2/4 proteins are crucial for offspring survival. Miscarriages were frequent and the offspring between maternal knockout females and heterozygous males of all three genotypes did not follow the expected 50:50 Mendelian ratio for the offspring heterozygous and homozygous null genotypes (Fig. 4A). Moreover, pup survival was severely impaired for both single and double knockouts, with a high lethality rate by P3 for offspring derived from maternal knockout females crossed with either WT or heterozygous males (Fig. 4B). Importantly, almost all offspring survived at similar rates to controls when WT females were crossed with heterozygous males (in which 50% of the offspring would be expected to be heterozygous), indicating that heterozygous offspring survive if they have a functional maternal allele of *Dppa2/4*.

Despite the high lethality, we were able to obtain three Dppa4^−/−^ adult males and two Dppa4^−/−^ females from a single litter from eight matings. No homozygous null animals survived weaning from *Dppa2* or *Dppa2/4* genotypes. We first assessed the fertility of the surviving adult Dppa4^−/−^ males and females by mating them to WT animals, from which they gave rise to viable offspring (Fig. 4C), indicating both male and female Dppa4^−/−^ animals were fertile. We then set up four separate matings between Dppa4^−/−^ males and Dppa4^m−^ females which had successfully given litters in other matings. Of these, two resulted in miscarriages and two produced litters with two offspring each which were stillborn or died immediately after birth (Fig. 4C).

We next sought to determine when during embryonic development this lethality occurred. We readily isolated similar numbers of Dppa4^−/−^ blastocysts as those from crosses between WT females and males (Fig. 4D). The Dppa4^−/−^ blastocysts did not show any morphological abnormalities, with proper cavity formation, similar number of blastomeres and correctly segregated CDX2^+^ trophectoderm and NANOG^+^ inner cell mass (Fig. 4E; Fig. S4A,B). Moreover, we were able to isolate normal looking E7.5 embryos from Dppa4^m−^×Dppa4^−/−^ crosses (Fig. 4D), a time at which Dppa2/4 are no longer expressed. Together, this suggests that the embryonic lethality and defects occur following implantation after *Dppa2/4* have been silenced. This is consistent with the findings from zygotic knockout mouse models (Madan et al., 2009; Nakamura et al., 2011), and a molecular function for Dppa2/4 as epigenetic priming factors in which they establish a permissive epigenome to facilitate future cell differentiation (Eckersley-Maslin et al., 2020; Gretarsson and Hackett, 2020).

Lastly, we disentangled the relative importance of maternal versus zygotic Dppa4 in embryonic survival. If only embryonic levels of Dppa4 were important for the development and survival of Dppa4^+/−^ animals, then it should not matter whether the mutant allele was inherited maternally or paternally. However, we only observed a marked impairment in the survival rate of Dppa4^+/−^ embryos when the mutant allele was inherited from the mother as opposed to the father (Fig. 4F). Therefore, maternal stores of Dppa4 are important for offspring survival, in addition to what is embryonically transcribed.

## DISCUSSION

Here, we systematically and comprehensively assess the importance of maternal Dppa2/4 using single and double conditional knockout mouse models. In contrast to predictions from *in vitro* studies, maternal Dppa2/4 is dispensable for ZGA and preimplantation development *in vivo*. However, the absence of maternal Dppa2/4 severely compromises development. Both maternal and zygotic Dppa2/4 are important for offspring survival and loss of maternal Dppa2/4 increases the severity of embryonic lethality in the homozygous embryos over what has been reported with zygotic knockout mouse models (Madan et al., 2009; Nakamura et al., 2011). No offspring that lack maternal and zygotic Dppa2 survive and the very rare offspring that lack both maternal and zygotic Dppa4 die shortly after birth. Importantly, heterozygous animals that inherit a null allele maternally fare worse than those that inherit the null allele paternally, indicating maternal stores of Dppa2/4 have key roles in development outside of ZGA.

The 2C-like cell *in vitro* system has widely been used to gain insights into the biology of two-cell embryos and ZGA (reviewed by Iturbide and Torres-Padilla, 2020). However, regulators of ZGA-associated transcripts *in vitro* do not always validate as ZGA regulators *in vivo*. Recently, the transcription factor Dux was also shown to be dispensable for ZGA *in vivo* (Bosnakovski et al., 2021; Chen and Zhang, 2019; De Iaco et al., 2020; Guo et al., 2019), despite being essential for the 2C-like state *in vitro* (De Iaco et al., 2017; Hendrickson et al., 2017). Therefore, although a useful tool, it is crucial that findings using the *in vitro* 2C-like cell system are validated *in vivo*.

In ESCs, Dppa2/4 function directly upstream of Dux to induce its expression (De Iaco et al., 2019; Eckersley-Maslin et al., 2019; Yan et al., 2019). Similar to our initial observations with Dppa2/4, *Dux* knockout mice also undergo successful ZGA yet have reduced litter sizes and heterozygous crosses deviate slightly from Mendelian frequencies (Bosnakovski et al., 2021; Chen and Zhang, 2019; De Iaco et al., 2020; Guo et al., 2019). However, in contrast to what we observe for Dppa2/4, *Dux* homozygous null animals are readily obtained, indicating that Dppa2/4 has additional roles in embryo development beyond regulating *Dux*.

Maternal knockouts of the majority of ZGA regulators lead to a delay and/or dampening of ZGA transcription, rather than a complete absence of ZGA in its entirety (reviewed by Eckersley-Maslin et al., 2018). One possibility is that there is a high degree of redundancy between ZGA regulators to ensure this key developmental milestone is reached and not leaving it vulnerable to a single mutation. Alternatively, defects in regulating ZGA may not always lead to major transcriptional changes in the two-cell embryo but may manifest more subtly with phenotypes only seen at later developmental or postnatal stages. Consequently, assessing phenotypes of maternal regulators of ZGA may be more complex than previously thought.

Importantly, our findings indicate that paternal and maternal Dppa2/4 are not equal. Although we failed to produce any Dppa2^−/−^ or Dppa2/4^−/−^ adult males, we obtained three rare Dppa4^−/−^ males from a single litter. These we used to disentangle the importance of the maternal versus paternal alleles in a context where embryos would all have the same genotype. Heterozygous Dppa4^+/−^ offspring that inherit a maternal null allele (from maternal knockout females) have much poorer survival rates than those that inherit a paternal null allele. It remains to be determined when during development the maternal stores are important. We were able to collect morphologically normal blastocysts, suggesting that the embryonic defects that lead to high mortality rate occur following implantation at a time when Dppa4 is no longer expressed, although we cannot rule out that the phenotypes are not a consequence of subtly affecting ZGA. We hypothesise that maternal Dppa2/4 may be bookmarking key developmental genes in the oocyte and zygote, priming them for activation at later developmental stages. Consistently, in ESCs Dppa2/4 function as priming factors and are required to establish bivalent chromatin, thus facilitating effective differentiation (Eckersley-Maslin et al., 2020; Gretarsson and Hackett, 2020). In support of a priming function in early embryos, we reveal that, both *in vitro* and *in vivo*, Dppa2/4 bind mitotic chromatin. This presents a mechanism by which Dppa2/4 could mark developmental promoters through the widespread epigenetic reprogramming that takes place in preimplantation embryos, ensuring they are appropriately activated in a timely manner at later developmental stages. Embryonically expressed Dppa2/4 may reinforce this priming function and partially compensate for lack of maternal stores. This uncoupling of when Dppa2/4 are present and the phenotypic consequences of their loss is a hallmark of epigenetic priming factors (Eckersley-Maslin, 2020). In this way, maternal proteins may function beyond ZGA and implantation to ensure the successful development of the embryo.

## MATERIALS AND METHODS

### Generation of conditional knockout mice

Conditional knockout mice were designed and generated by Cyagen. Homology arms were amplified by BAC clones and introduced into targeting vectors containing a Neomycin selection cassette flanked by LoxP sites. For *Dppa2* and *Dppa4* single knockouts, exon 2 was selected as the conditional knockout region. For the double *Dppa2/4* conditional knockout, exon 2 of *Dppa4* through to exon 7 of *Dppa2* was selected as the conditional knockout region. Targeted C57Bl/6 mouse ESCs were identified by genotyping PCR and confirmed by Southern Blot. Targeted ESCs were injected into C57Bl/6 albino embryos, which were re-implanted into CF-1 pseudo-pregnant females. Founder animals were identified by their coat colour and germline transmission confirmed by breeding with Flp-deleter females and subsequent genotyping of the offspring. Conditional knockout lines were maintained by intercrossing conditional homozygous (c/c) or heterozygous (c/+) animals. To generate maternal knockout mice, female c/c mice on a C57Bl/6 background were crossed with Zp3-Cre males (Lewandoski et al., 1997). All experimental procedures were performed in accordance with the Animals (Scientific Procedures) Act 1986 and by local authority granted by the Animal Welfare and Ethical Review Body (AWERB) committee of the Babraham Institute, UK.

### Embryo collection

All mouse embryos were collected from natural matings between the appropriate genotypes according to standard procedures (Hogan, 1994), at different time points, depending on the desired stage. MII oocytes were collected from C57Bl/6 females in oestrus, and zygotes, two-cell embryos, four-cell embryos, eight-cell embryos, morula and blastocysts at E0.5, E1.5, E2, E2.5, E3 and E3.5, respectively. Embryos used in this study were derived from a cross of C57Bl/6 females mated with C57Bl/6 males. The knockout embryos were derived from the maternal knockout females mated with either C57Bl/6 proven studs or the appropriate genotype (*Dppa2*, *Dppa4*, *Dppa2/4*^+/−^ or *Dppa4*^−/−^) males.

### Mouse embryonic stem cell culture

E14 mouse ESCs were cultured in serum/LIF conditions (DMEM, 45,000 mg/l glucose, 4 mM L-glutamine, 110 mg/l sodium pyruvate, 15% fetal bovine serum, 1 U/ml penicillin, 1 mg/ml streptomycin, 0.1 mM nonessential amino acids, 40 mM b-mercaptoethanol, 10^3^ U/ml LIF). Plasmids containing full length Dppa2-GFP and Dppa4-GFP were generated previously (Eckersley-Maslin et al., 2019). Truncations were generated by amplifying appropriate sections of the constructs and cloning into the pDONR221 vector. Gateway cloning was then used to transfer the truncated cDNA sequences into an in-house built pDEST vector as previously described (Eckersley-Maslin et al., 2019). Plasmids were transfected into E14 ESCs using Lipofectamine 2000.

### Immunofluorescence staining

Oocytes and embryos were washed in PBS, fixed for 15 min in 4% paraformaldehyde in PBS, permeabilised with 0.5% Triton X-100 in PBS for 1 h and blocked in 1% bovine serum albumin (BSA) in PBS for 1 h. Primary antibodies were diluted in 1% BSA and the embryos incubated for 1 h. After 1 h wash in 1% BSA, the samples were incubated in secondary antibodies for 45 min, followed by a 1 h wash in PBT (0.05% Tween 20 in PBS). DNA was counterstained with 5 μg/ml DAPI in PBS, and embryos mounted in fibrin clots. All incubations were performed at room temperature. Primary antibodies and dilutions used were: mouse anti- Dppa2 (Millipore, mab4356, 1:100), goat anti- Dppa4 (R&D Systems, AF3730, 1:100), rabbit anti-MERVL (Huabio, R1501-2, 1:200), mouse anti-CDX2 (Biogenex, MU392-UC, 1:200), rabbit anti-NANOG (Abcam, ab80892, 1:200). Secondary antibodies used were: anti-rabbit AF-conjugated 647, anti-mouse AF-conjugated 568 and anti-goat AF-conjugated 488 (Molecular Probes, A31573, A10037, A11055, 1:1000). Single optical sections and *z*-stacks were captured using a Zeiss LSM780 microscope (63× oil-immersion objective). Fluorescence colocalisation analysis was performed with ImageJ, and fluorescence intensity measurements were performed with Volocity 6.3 (Improvision). The nuclei of each blastomere in the two-cell embryos were measured separately and their average was used for the final analysis. The plots were generated using RStudio.

### Single-cell RNA-seq

Embryos from the Dppa2/4^m−^ were generated from natural matings between males and females of relevant genotypes. The zona pellucida was removed using Tyrode's solution (Sigma-Aldrich, T1788) and individual embryos were placed in in 2.5 µl methyltransferase reaction mixture, according to the published protocol (Clark et al., 2018). mRNA was captured using Smart-seq2 oligo-dT pre-annealed to magnetic beads (MyOne C1, Invitrogen). The lysate containing the gDNA was transferred to a separate PCR plate and the beads were washed three times in 15 ml FSS buffer (Superscript II, Invitrogen), 10 mM DTT, 0.005% Tween 20 (Sigma-Aldrich) and 0.5 U/ml of RNAsin (Promega). The beads were then resuspended in 10 ml of reverse transcriptase mastermix [100 U SuperScript II (Invitrogen), 10 U RNAsin (Promega), 1× Superscript II First-Strand Buffer, 2.5 mM DTT (Invitrogen), 1 M Betaine (Sigma-Aldrich), 9 mM MgCl2 (Invitrogen), 1 mM Template-Switching Oligo (Exiqon), 1 mM dNTP mix (Roche)] and incubated on a thermocycler for 60 min at 42°C, followed by 30 min at 50°C and 10 min at 60°C. PCR was then performed by adding 11 ml of 2× KAPA HiFi HotStart ReadyMix and 1 ml of 2 mM ISPCR primer, and cycling as follows: 98°C for 3 min, 15 cycles of 98°C for 15 s, 67°C for 20 s, 72°C for 6 min and finally 72°C for 5 min. cDNA was purified using a 1:1 volumetric ratio of Ampure Beads (Beckman Coulter) and eluted in 20 ml of water. Libraries were prepared from 100 to 400 pg of cDNA using Nextera XT Kit (Illumina), as per the manufacturer's instructions but with one-fifth volumes for each sample. Libraries were sequenced on an Illumina NextSeq500 MidOutput 75 bp paired-end reads per embryo. Data are available at GEO under accession number GSE184763.

### RNA-seq analysis

Libraries were trimmed using Trim Galore (v0.6.5, Cutadapt v2.3) and mapped to the mouse GRCm38 genome assembly using HISAT2 (v2.1.0, --no-softclip) and filtered to have MAPQ scores of 20 and above. Data were quantified using the RNA-seq quantitation pipeline in SeqMonk (https://www.bioinformatics.babraham.ac.uk/projects/seqmonk/), without strand-specific quantification, using mRNA probes. All the analysis, specified in each figure legend, was carried in SeqMonk, and only the violin plots were generated in RStudio. The major ZGA and maternal deposited gene lists used in the analysis were generated using the publicly available dataset (GSE44183) from Xue et al. (2013). The 2C-like gene list was from Eckersley-Maslin et al. (2016).

## Supplementary Material

Supplementary information

Reviewer comments
