# Supplementary material for: Maternal Dppa2 and Dppa4 are dispensable for zygotic genome activation but important for offspring survival
Source: Development. 2021 Dec 21;148(24):dev200191. doi: 10.1242/dev.200191 (PMC8722389; doi:10.1242/dev.200191)
Supplement: Supplementary information [file develop-148-200191-s1.pdf]

Kubinyecz et al. Supplemental Figure 1

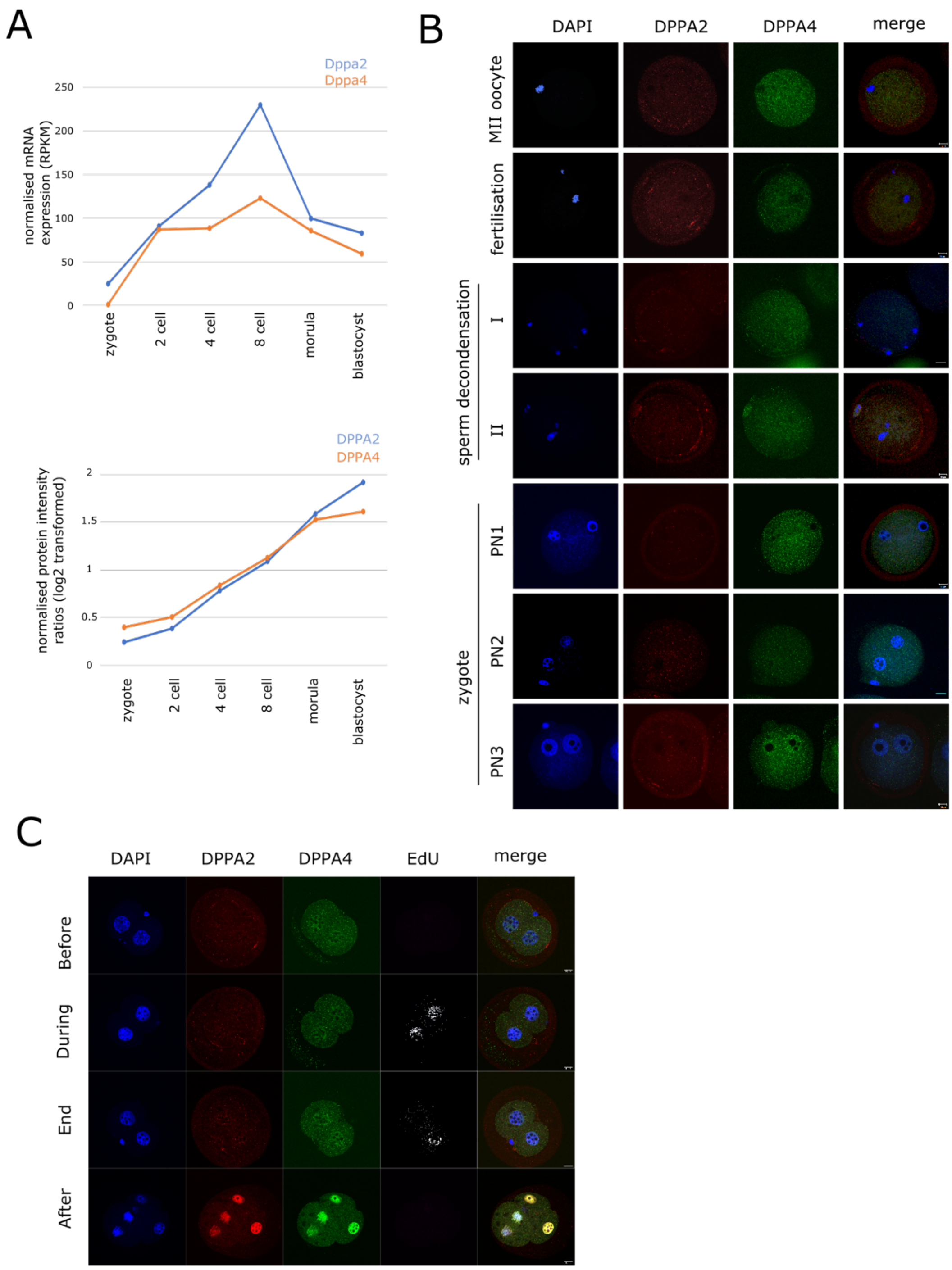

Fig. S1. Dppa2/4 localise to euchromatin in preimplantation embryos and associate with mitotic chromatin. Related to Figure 1.

(A) transcript (top) and protein (bottom) levels of Dppa2 (blue) and Dppa4 (orange) during preimplantation development as measured in Guo et al. 2017. (B) Immunofluorescence staining of DPPA2 (red) and DPPA4 (green) in MII oocytes and zygotes of different stages. Scale bar represents 10µm. (C) Immunofluorescence staining of DPPA2 (red) and DPPA4 (green) in 2-cell embryos undergoing DNA replication. EdU (grey) labels newly synthesised DNA. Stage of DNA replication is denoted. Scale bar 10µm.

Development • Supplementary information

Kubinyecz et al. Supplemental Figure 2

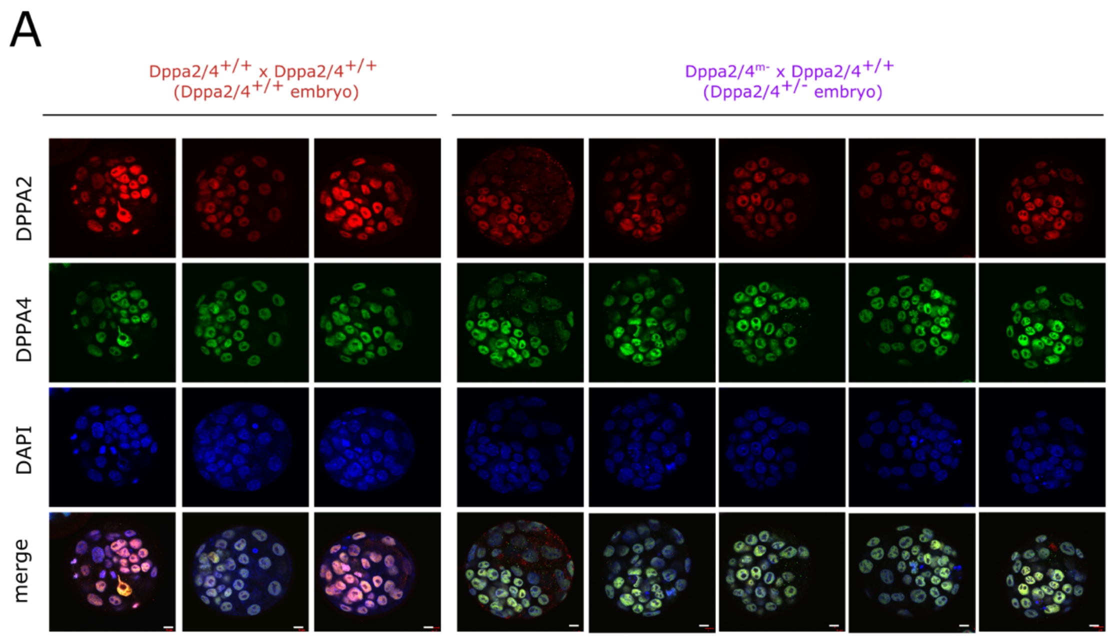

Fig. S2. Maternal Dppa2/4 single and double knockout mice are fertile. Related to Figure 2.

(A) Immunofluorescence staining of blastocysts collected from Dppa2/$4^{+/+}$ (left) or Dppa2/4^{m-} (right) females crossed with Dppa2/$4^{+/+}$ fathers for DPPA2 (top, red), DPPA4 (second row, green) and DAPI nuclear stain (third row, blue). Scale bar 10µm.

Development • Supplementary information

Kubinyecz et al. Supplemental Figure 3

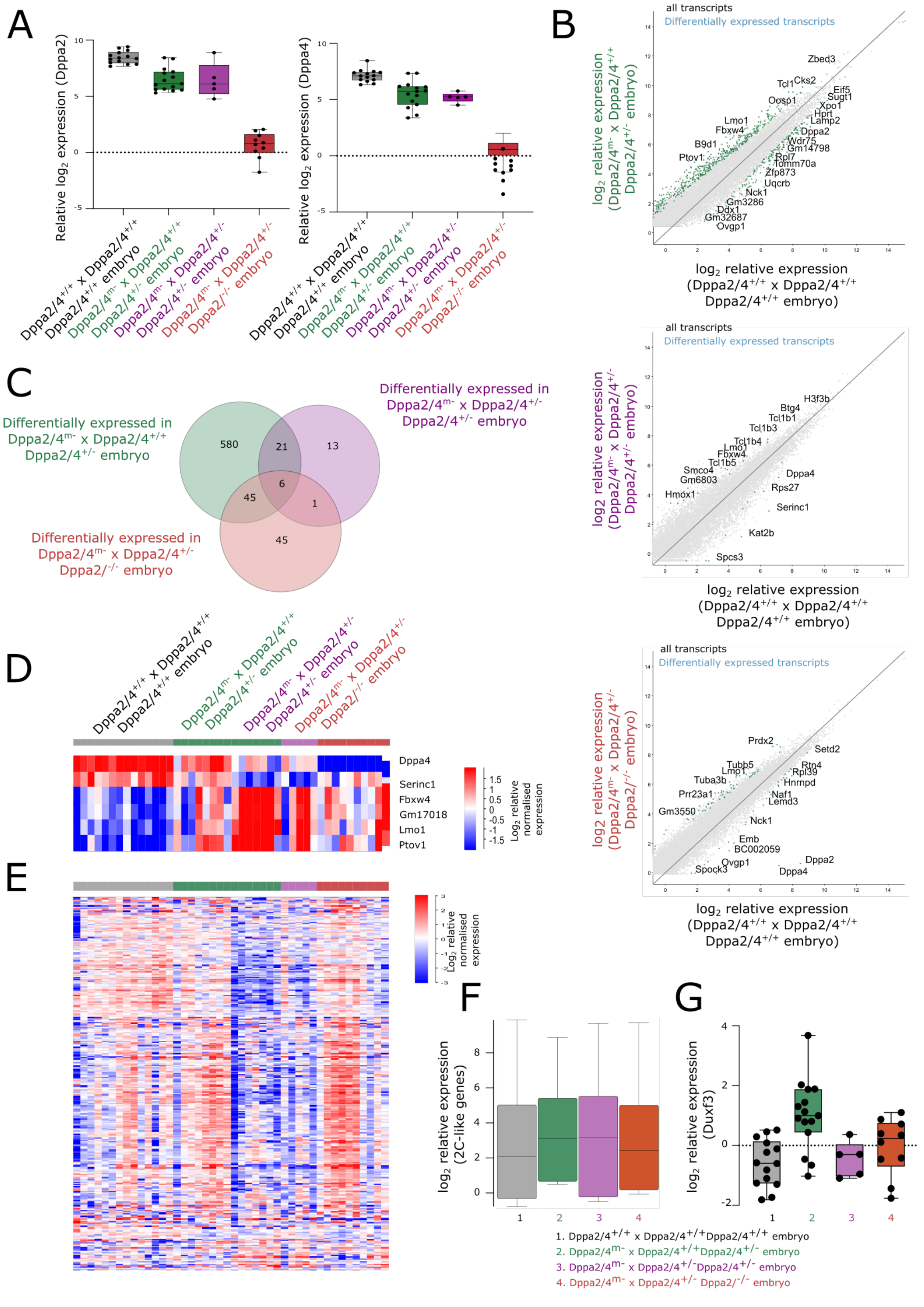

Development • Supplementary information

Fig. S3. Two-cell embryos from maternal knockout females undergo successful ZGA with subtle differences. Related to Figure 3.

(A) Relative expression of Dppa2 (left) and Dppa4 (right) in 2-cell embryos from Dppa2/4$^{+/+}$ x Dppa2/4$^{+/+}$ (embryos Dppa2/4$^{+/+}$); Dppa2/4^{m-} x Dppa2/4$^{+/+}$ (embryos Dppa2/4$^{+/-}$); Dppa2/4^{m-} x Dppa2/4$^{+/-}$ (embryos Dppa2/4$^{+/-}$ or Dppa2/4$^{-/-}$). (B) Scatter plots showing differential expressed genes (blue) between wild type embryos and Dppa2/4$^{+/-}$ or Dppa4$^{-/-}$ embryos from different crosses. (C) Venn diagram showing overlap in differentially expressed genes between the different comparisons in B. (D) Per probe normalised heatmap of the 6 differentially expressed genes consistently changed in all three comparisons. (E) Per probe normalised heatmap showing relative expression of major ZGA transcripts in 2-cell embryos. Samples are clustered by genotype. (F) box-whisker plot showing expression levels of 2C-like genes in the different genotypes. Gene list from (Eckersley-Maslin et al. 2016). (G) Expression of Duxf3 in individual embryos from each of the different genotypes.

Kubinyecz et al. Supplemental Figure 4

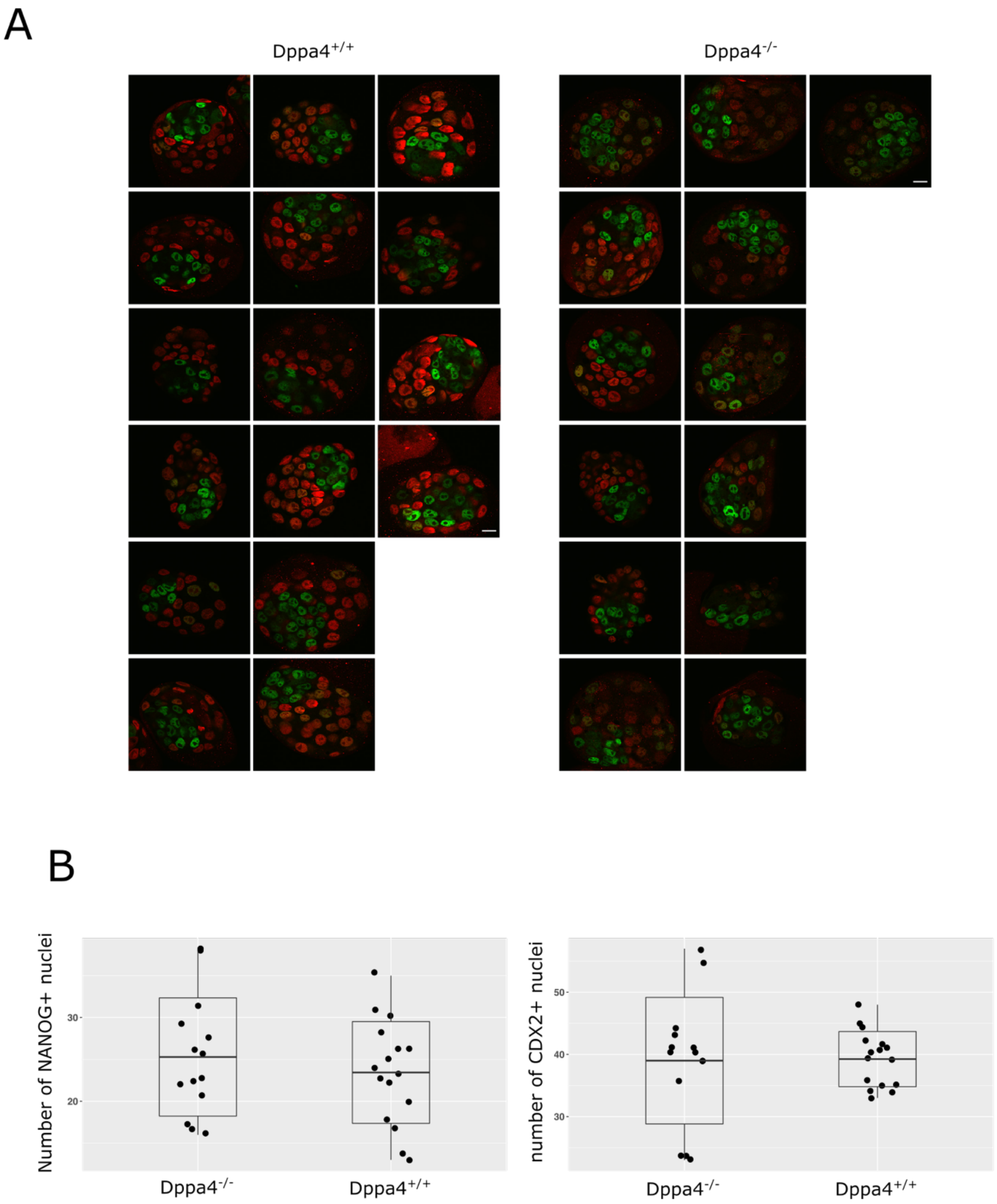

Fig. S4. Both maternal and zygotic Dppa4 are required for offspring survival. Related to Figure 4.

(A) Immunofluorescence staining of NANOG (green) and CDX2 (red) in E3.5 $Dppa4^{+/+}$ (left) or $Dppa4^{-/-}$ (right) blastocysts. Scale bar represent 10µm. (B) Number of NANOG+ and CDX2 + blastomeres in $Dppa4^{+/+}$ or $Dppa4^{-/-}$ E3.5 blastocysts.

Development • Supplementary information

Supplemental Materials and Methods - Generation of conditional knockout mice

Dppa2 single conditional knockout mouse model

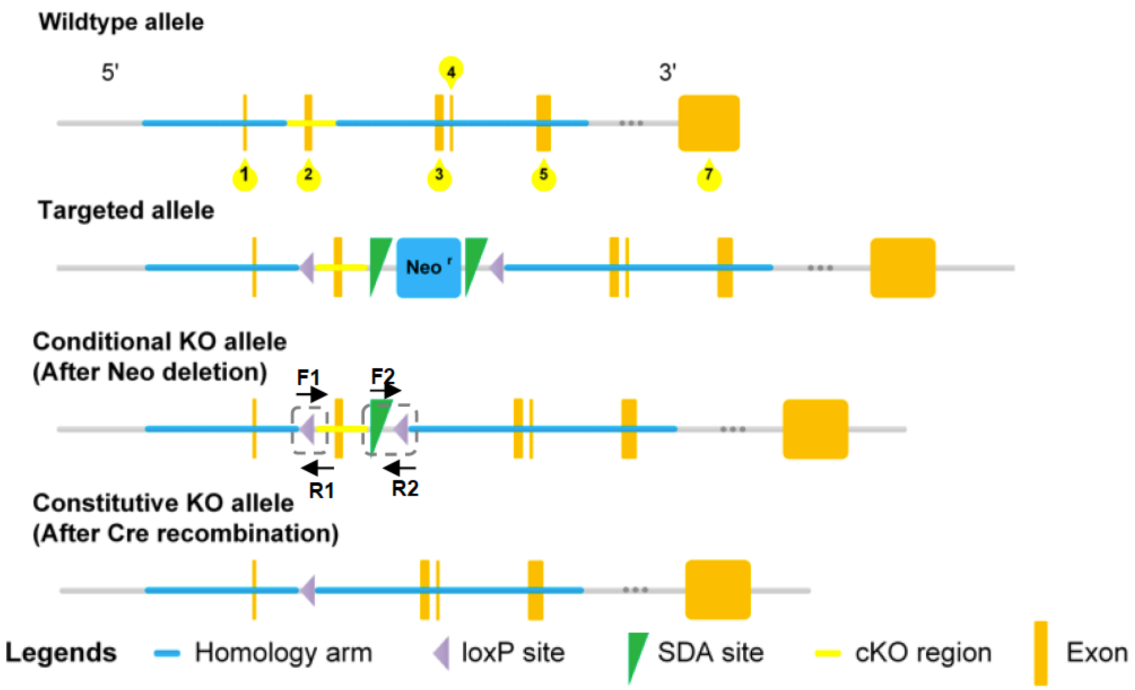

Primers for LoxP PCR
loxP-F (F1): 5'-CCACTAGCCTGTCAATATTGCTTTT-3'
loxP-R (R1): 5'-GACACAAATCCACTTCTGTTACCAT-3'
Expected PCR Product: Wildtype: 300 bp; Targeted: 356 bp

Primers for Neo deletion PCR
Neo-del-F (F2): 5'-TCAGTTGTTAAGCCCTGTGAACT-3'
Neo-del-R (R2): 5'-GGCTTTTGCTACCATGTCCTTTT-3'
Expected PCR product: Wildtype: 323bp; Targeted: 436bp

Primers for targeted allele:
Neo-del-F (F2): 5'-TCAGTTGTTAAGCCCTGTGAACT-3'
Neo-del-R (R2): 5'-GGCTTTTGCTACCATGTCCTTTT-3'
Wildtype: 323 bp; Homozygotes: 436 bp; Heterozygotes: 436 bp/323 bp

Primers for Cre transgene:
Forward1: 5'- ACCACTAGCCTGTCAATATTGCTT-3'
Reverse1: 5'- GGCTTTTGCTACCATGTCCTTTTA-3'
Cre amplicon: 413 bp

Primers for constitutive KO allele:
cKO-F (F3): 5'- ACCACTAGCCTGTCAATATTGCTT-3'
cKO-R (R3): 5'- GGCTTTTGCTACCATGTCCTTTTA-3'

Development • Supplementary information

Constitutive KO allele: 475 bp

Dppa4 single conditional knockout mouse model

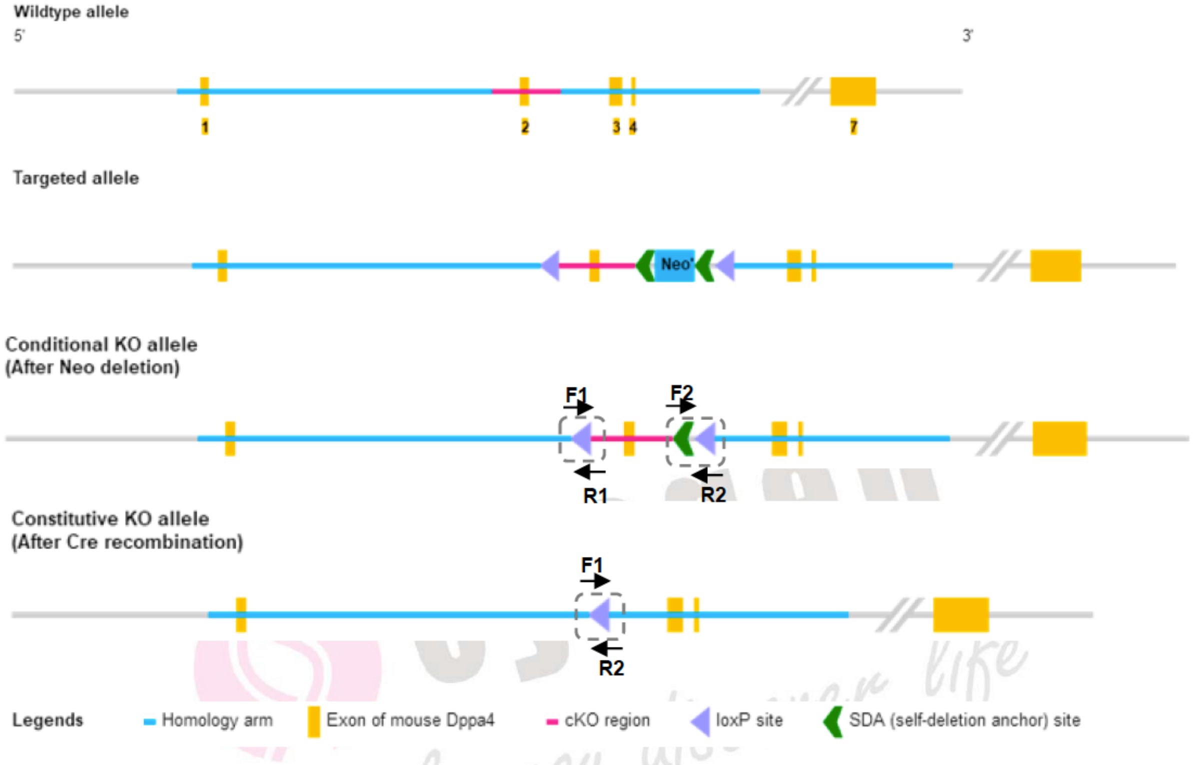

Primers for LoxP PCR
loxP-F (F1): 5'-TGTGCCCATATCTTCGAGGCTTTCC-3'
loxP-R (R1): 5'-AGAGCATCATCTGAATAACCAACCTG-3'
Expected PCR product: Wildtype 329bp, Targeted 385bp

Primers for Neo deletion PCR:
Neo-del-F (F2): 5'-CCTAGCTGGGTTCTTACCTCATTAAA-3'
Neo-del-R (R2): 5'-AACAAGTGCAAACTGTCCTCATTG-3'
Expected PCR Product: Wildtype: 181 bp; Targeted: 300 bp

Primers for targeted allele:
Neo-del-F (F2): 5'-CCTAGCTGGGTTCTTACCTCATTAAA-3'
Neo-del-R (R2): 5'-AACAAGTGCAAACTGTCCTCATTG-3'
Wildtype: 181 bp; Homozygotes: 300 bp; Heterozygotes: 300 bp/181 bp

Primers for Cre transgene:
Forward1: 5'-CATATTGGCAGAACGAAAACGC-3'
Reverse1: 5'-CCTGTTTCACTATCCAGGTTACGG-3'
Cre amplicon: 413 bp

Primers for constitutive KO allele:
loxP-F (F1): 5'-TGTGCCCATATCTTCGAGGCTTTCC-3'
Neo-del-F (F2): 5'-CCTAGCTGGGTTCTTACCTCATTAAA-3'

Development • Supplementary information

Neo-del-R (R2): 5’-AACAAGTGCAAACTGTCCTCATTG-3’
Wildtype allele: 181 bp; Conditional KO allele: 300 bp; Constitutive KO allele: 216 bp

Dppa2/4 double conditional knockout mouse model

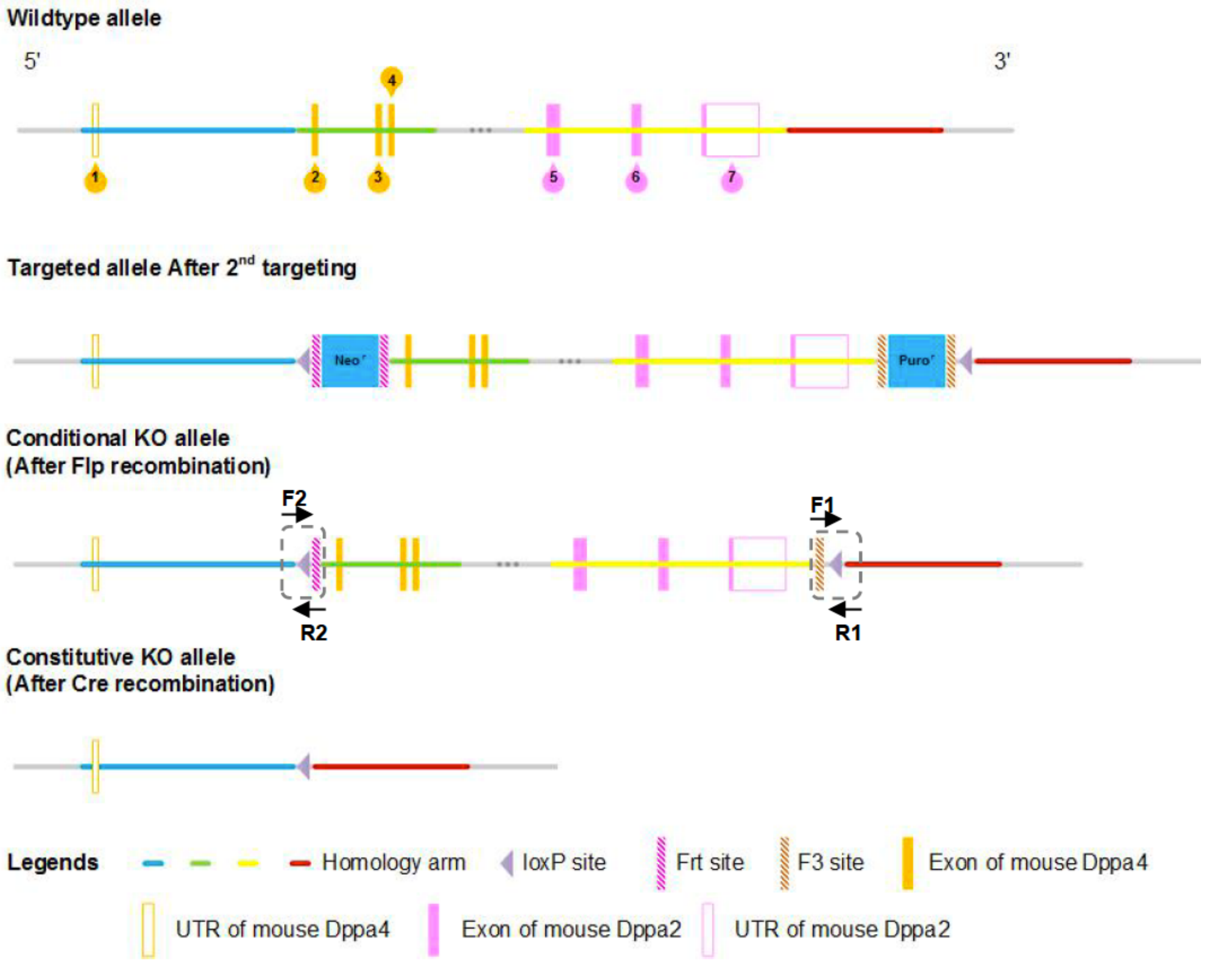

Primers for Puro deletion PCR:
Puro-del-F (F1): 5’-CTTGGAACTTCTGAGCCCCCCC-3’
Puro-del-R (R1): 5’-GGGCTGAGGTAGAATTAGTTCC-3’
Expected PCR Product: Wildtype: 220 bp; Targeted: 352 bp

Primers for Neo deletion PCR:
Neo-del-F (F2): 5’-ATGTCACAGCGCAAGCCATTG-3’
Neo-del-R (R2): 5’-GGCAACATAAAAAACAGGCTCA-3’
Expected PCR Product: Wildtype: 163 bp; Targeted: 291 bp

Primers for targeted allele:
Neo-del-F (F2): 5’-ATGTCACAGCGCAAGCCATTG-3’
Neo-del-R (R2): 5’-GGCAACATAAAAAACAGGCTCA-3’
Wildtype: 163 bp; Homozygotes: 291 bp; Heterozygotes: 291 bp/163 bp

Primers for Cre transgene:

Development • Supplementary information

Forward1: 5’-CATATTGGCAGAACGAAAACGC-3’
Reverse1: 5’-CCTGTTTCACTATCCAGGTTACGG-3’
Cre amplicon: 413 bp

Primers for constitutive KO allele:
Neo-del-F (F2): 5’-ATGTCACAGCGCAAGCCATTG-3’
Neo-del-R (R2): 5’-GGCAACATAAAAAACAGGCTCA-3’
Wildtype allele: 163 bp; Conditional KO allele: 291 bp

Neo-del-F (F2): 5’-ATGTCACAGCGCAAGCCATTG-3’
Puro-del-R (R1): 5’-GGGCTGAGGTAGAATTAGTTCC-3’
Constitutive KO allele: 401 bp

Table S1. Gene lists and expression values relating to Figure 3.

Click here to download Table S1
