# Supplementary material for: Maternal Dppa2 and Dppa4 are dispensable for zygotic genome activation but important for offspring survival
Source: Development. 2021 Dec 21;148(24):dev200191. doi: 10.1242/dev.200191 (PMC8722389; doi:10.1242/dev.200191)
Supplement: Reviewer comments [file develop-148-200191_review_history.pdf]

Maternal Dppa2 and Dppa4 are dispensable for zygotic genome activation but important for offspring survival

Oana Kubinyecz, Fatima Santos, Deborah Drage, Wolf Reik and Melanie A Eckersley-Maslin
DOI: 10.1242/dev.200191

Editor: Liz Robertson

Review timeline
Original submission: 14 September 2021
Editorial decision: 4 October 2021
First revision received: 26 October 2021
Accepted: 22 November 2021

Original submission

First decision letter

MS ID#: DEVELOP/2021/200191

MS TITLE: Maternal Dppa2 and Dppa4 are dispensable for zygotic genome activation but important for offspring survival

AUTHORS: Oana Kubinyecz, Fatima Santos, Deborah Drage, Wolf Reik, and Melanie A Eckersley-Maslin

I have now received all the referees reports on the above manuscript, and have reached a decision. The referees' comments are appended below, or you can access them online: please go to BenchPress and click on the 'Manuscripts with Decisions' queue in the Author Area.

The overall evaluation is positive and we would like to publish a revised manuscript in Development, provided that the referees' comments can be satisfactorily addressed. Please attend to all of the reviewers' comments in your revised manuscript and detail them in your point-by-point response. If you do not agree with any of their criticisms or suggestions explain clearly why this is so.

We are aware that you may currently be unable to access the lab to undertake experimental revisions. If it would be helpful, we encourage you to contact us to discuss your revision in greater detail. Please send us a point-by-point response indicating where you are able to address concerns raised (either experimentally or by changes to the text) and where you will not be able to do so within the normal timeframe of a revision. We will then provide further guidance. Please also note that we are happy to extend revision timeframes as necessary.

Reviewer 1

Advance summary and potential significance to field

The major wave of zygotic genome activation (ZGA) in mice begins at the 2-cell stage and is essential for early embryo development. 2C-like ES cells represent a small subpopulation of mouse embryonic stem (ES) cells and share some genetic and epigenetic features with mouse 2-cell embryos. Results from 2C-like ES cells have been used as proxies for 2-cell

© 2021. Published by The Company of Biologists under the terms of the Creative Commons Attribution License (https://creativecommons.org/licenses/by/4.0/).

embryos. Developmental Pluripotency Associated 2 (Dppa2) and 4 (Dppa4) genes were previously identified activators of Dux expression and potent inducers of the 2C-like state. Their ablation affects differentiation of ES cells in vitro.

Comments for the author

Using Zp3-Cre and Dppa2/4 floxed mouse lines, the authors established single cKO Dppa2, Dppa4 as well as a double cKO mouse lines. Experiments with these lines documented that Dppa2/4 were not required for pre-implantation development and did not affect ZGA but did affect offspring survival.
Suggestions for the authors to consider:
1. Rules for capitalization and italization of genes and proteins should be adhered to in the text and images. For example, in Fig. S3A legend and the y-axis in Fig. S3B, proteins should be capitalized (NANOG - not Nano; CDX-2) and genes should be italicized (Dppa2/Dppa4).
2. Line 99: does the phrase "transcriptional upregulation during the major wave of ZGA" refer to Dppa2/4 and, if so, can it be documented with a plot in Fig. S1 from cited scRNA-seq data of early embryos.
3. Fig. 1B: DPPA2 and DPPA4 can be detected in mitotic and non-mitotic cells. However, the authors emphasize DPPA2/4 association with mitotic chromatin. While DPPA2/4 appears to bind to euchromatin in the figures could it also bind to other regions?
4. Fig. 1C: Please provide DAPI staining and DPPA2/4 staining in separated panels together with merged images. Each image should contain both mitotic and non-mitotic cells.
5. Fig. 2C: DPPA2/4 in embryos should be co-stained with antibodies to NANOG (or other ICM cell markers) and CDX-2.
6. Fig. 2D: Statistical analyses would be improved by more samples (litters), e.g., Dppa2 allele (+/+ x +/+; m- x +/+; +/+ x +/-) and Dppa2/4 allele (+/+ x +/+). The Dppa4 allele plot of m- x +/- does not appear complete.
7. Fig. 2D: The authors conclude that Dppa2m- and Dppa4m- had smaller litters. However,
a. Significant decreases in litter sizes were only observed in Dppa2m- x +/-, Dppa4m- x +/- and Dppa2/4m- x +/- groups which is consistent with significant defects in Dppa2-/-, Dpp4-/- and Dpp2/4-/- pups.
b. Litter sizes of Dppa2/4 m- x +/+ mating were not decreased compared to +/+ x +/+ mating.
c. Dppa2m- x +/+ had a slight (not statistically significant) decrease in litter size compared to +/+ x +/+.
d. Dppa4m- x +/+ had a slight (not statistically significant) decrease in litter size compared to +/+ x +/+.
e. The results from b-d make it difficult to conclude "In the case of Dppa2m- and Dppa4m-, the resulting litter sizes were smaller". Statistical analysis from more litters may provide more solid evidence.
8. In Fig. 3A, the localization of MuERV-L-Gag appears different from that in the paper published by Macfarlan et. al. (2012). Is this observation correct?
9. In Fig. 3E, it is difficult to read the genotype of each column in the heatmap. Placing embryos with same genotype in adjacent columns may make it easier to read or the figure could be larger in supplementary information.
10. In Fig. 3F, please indicate how many different genes in each group are used. The gene lists (maternal, MERVL and major ZGA transcripts) should be provided as supplementary tables.
11. Line 187: It seems to be safe to conclude that "neither maternal nor zygotic Dppa2/4 is essential for ZGA in vivo" to better summarize the results of Fig. 3C-3F.
12. Fig. 4B: Is the color scheme of the middle panel correct?
13. Fig. 4C and 4D: More samples should be included to make the analysis robust.
14. Lines 205-212: The fertility of Dppa4-/- females should be discussed.
15. Fig. S2B: The font size of gene labels should be increased.
16. Line 241: the authors mentioned "maternal DPPA4 is important for offspring survival, and the complete absence of both maternal and embryonic DPPA4 is not compatible with development." Is DPPA4 correct? It appears that some Dppa4-/- adults were obtained, but no Dppa2-/- adults 17. Careful copy-editing throughout would improve the manuscript. For example, there are multiple incomplete/errors in References.

© 2021. Published by The Company of Biologists under the terms of the Creative Commons Attribution License (https://creativecommons.org/licenses/by/4.0/).

Reviewer 2

Advance summary and potential significance to field

In this study, the authors performed a vey comprehensive experiment and proved that the maternal deletion of both Dppa2 and Dppa4 showed no obvious effect on the zygotic genome activation. The experiments were well designed and conducted, and the results are important for the field to re-consider the important regulators of ZGA in mammals. The manuscript would be acceptable after minor revision.

Comments for the author

Major concern:
In the previous study from the group has shown that Dppa2 and Dppa4 can activate Dux in 2-cell like ES cells. In the double CKO embryos, the authors only checked the expression of MERVL and if the expression of Dux is affected? The authors can present this result and give some interpretation in terms the difference from ES studies.

First revision

Author response to reviewers' comments

We thank the reviewers for their positive comments on the revised manuscript. We have addressed all the comments below in a point by point rebuttal and incorporated the changes into the revised manuscript where appropriate.

Reviewer 1 Advance Summary and Potential Significance to Field:

The major wave of zygotic genome activation (ZGA) in mice begins at the 2-cell stage and is essential for early embryo development. 2C-like ES cells represent a small subpopulation of mouse embryonic stem (ES) cells and share some genetic and epigenetic features with mouse 2-cell embryos. Results from 2C-like ES cells have been used as proxies for 2-cell embryos. Developmental Pluripotency Associated 2 (Dppa2) and 4 (Dppa4) genes were previously identified activators of Dux expression and potent inducers of the 2C-like state. Their ablation affects differentiation of ES cells in vitro.

Reviewer 1 Comments for the Author:

Using Zp3-Cre and Dppa2/4 floxed mouse lines, the authors established single cKO Dppa2, Dppa4 as well as a double cKO mouse lines. Experiments with these lines documented that Dppa2/4 were not required for pre-implantation development and did not affect ZGA but did affect offspring survival.

Suggestions for the authors to consider:
1. Rules for capitalization and italization of genes and proteins should be adhered to in the text and images. For example, in Fig. S3A legend and the y-axis in Fig. S3B, proteins should be capitalized (NANOG - not Nano; CDX-2) and genes should be italicized (Dppa2/Dppa4).
We have gone through the manuscript carefully to ensure we adhere to the convention for naming genes/mRNA/proteins

2. Line 99: does the phrase “transcriptional upregulation during the major wave of ZGA” refer to Dppa2/4 and, if so, can it be documented with a plot in Fig. S1 from cited scRNA-seq data of early embryos.
Yes, this line is referring to the transcriptional upregulation of Dppa2/4. We have added a plot in the supplemental to support this as suggested (new Supplemental Figure 1A) and clarified this in the text (see lines 97-98).

© 2021. Published by The Company of Biologists under the terms of the Creative Commons Attribution License (https://creativecommons.org/licenses/by/4.0/).

3. Fig. 1B: DPPA2 and DPPA4 can be detected in mitotic and non-mitotic cells. However, the authors emphasize DPPA2/4 association with mitotic chromatin. While DPPA2/4 appears to bind to euchromatin in the figures,
could it also bind to other regions?
The euchromatic localisation of Dppa2/4 has been previously described (Masaki 2007, Masaki 2010, Nakamura 2011) and is consistent with our observations in non-mitotic cells where the majority of Dppa2/4 is euchromatic. We have clarified this in the text (see lines 103-104) and stated that DPPA2/4 protein is predominantly euchromatic because at this resolution we cannot rule out low level of binding to other regions.

4. Fig. 1C: Please provide DAPI staining and DPPA2/4 staining in separated panels together with merged images. Each image should contain both mitotic and non-mitotic cells.
We have done this as the reviewer has suggested and have also added arrows to clearly show the mitotic cells.

5. Fig. 2C: DPPA2/4 in embryos should be co-stained with antibodies to NANOG (or other ICM cell markers) and CDX-2.
Unfortunately, we had to shut down our Dppa2/4 conditional mouse colonies due to COVID-19 related restrictions and so are unable to collect more blastocysts to do these stains. We have, however, included additional blastocysts in supplemental figure 2A so that the reader can be reassured that the maternal knockout derived blastocysts are produced and phenotypically normal. We have also removed the statement in the results where we talk about different levels between ICM and trophectoderm as we do not have the staining to confirm the identity of the blastomeres.

6. Fig. 2D: Statistical analyses would be improved by more samples (litters), e.g., Dppa2 allele (+/+ x +/+; m- x +/+; +/+ x +/-) and Dppa2/4 allele (+/+ x +/+). The Dppa4 allele plot of m- x +/- does not appear complete.
As mentioned above, unfortunately we had to shut down the Dppa2/4 CKO mouse colonies due to limited staffing in our animal unit during the COVID19 lockdown, and so are unable to generate more matings. We have replotted the data to spread the datapoints out and included the number of litters analysed as during figure preparation some of the data points overlapped giving the impression that fewer litters were analysed than were in reality. The number of litters analysed is in line with that reported for Dux maternal knockout mice (Chen and Zhang, 2019 Nat Genet), and appropriate statistics have been implemented given the small sample size. Importantly more samples will not change the important conclusion from this analysis that the maternal knockout mice are fertile.

7. Fig. 2D: The authors conclude that Dppa2m- and Dppa4m- had smaller litters. However,
a.Significant decreases in litter sizes were only observed in Dppa2m- x +/-, Dppa4m- x +/- and Dppa2/4m- x +/- groups which is consistent with significant defects in Dppa2-/-, Dpp4-/- and Dpp2/4-/- pups.
b.Litter sizes of Dppa2/4 m- x +/+ mating were not decreased compared to +/+ x +/+ mating.
c.Dppa2m- x +/+ had a slight (not statistically significant) decrease in litter size compared to +/+ x +/+.
d.Dppa4m- x +/+ had a slight (not statistically significant) decrease in litter size compared to +/+ x +/+.
e.The results from b-d make it difficult to conclude “In the case of Dppa2m- and Dppa4m-, the resulting litter sizes were smaller”. Statistical analysis from more litters may provide more solid evidence.
As mentioned above in our response to point 7 unfortunately the Dppa2/4 CKO colonies have been shut down and so we are unable to generate more matings and analyse more litters. We have changed the message in the text to focus on the main conclusion from this data (that the maternal knockout mice give rise to live offspring) and suggest there may be an effect on litter size but more litters would need to be analysed to fully support this conclusion (see lines 138-141). We have also removed this statement from the abstract.

8. In Fig. 3A, the localization of MuERV-L-Gag appears different from that in the paper published by Macfarlan et. al. (2012). Is this observation correct?
In our manuscript we present a single z-slice section that shows a predominantly cytoplasmic localisation of MERVL-Gag protein. The figure in Macfarlan 2012 appears to be a maximum

© 2021. Published by The Company of Biologists under the terms of the Creative Commons Attribution License (https://creativecommons.org/licenses/by/4.0/).

intensity projection rather than a single z-slice. Indeed when we do a maximum intensity projection on our image stacks the image is very similar (see figure below). We have clarified that the figure in the manuscript is a single z-slice in the figure legend to avoid any confusion.

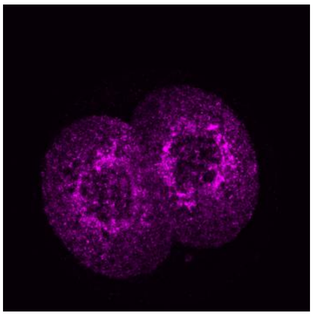

Maximum intensity projection of MERVL-GAG staining in a wild type 2-cell embryo. Note that now there is signal overlapping where the nuclei would be as a consequence of the projection.

9. In Fig. 3E, it is difficult to read the genotype of each column in the heatmap. Placing embryos with same genotype in adjacent columns may make it easier to read or the figure could be larger in supplementary information.
We intentionally clustered the embryos in this heatmap to highlight that they do not segregate by genotype. However, we understand the difficulty in reading the genotypes so have a) adjusted the colours to make this clearer, b) increased the size of this plot in Figure 3E, and c) included a heatmap in the supplement ordered by genotype.

10. In Fig. 3F, please indicate how many different genes in each group are used. The gene lists (maternal, MERVL and major ZGA transcripts) should be provided as supplementary tables.
We have indicated numbers of transcripts in each category in Figure 3F and include gene lists along with expression values for each individual embryo as a supplemental table as suggested, along with gene lists and expression values for the differentially expressed genes.

11. Line 187: It seems to be safe to conclude that “neither maternal nor zygotic Dppa2/4 is essential for ZGA in vivo” to better summarize the results of Fig. 3C-3F.
We will adjust the text to state this as suggested (now lines 188-189).

12. Fig. 4B: Is the color scheme of the middle panel correct?
Yes it is correct.

13. Fig. 4C and 4D: More samples should be included to make the analysis robust.
While we were able to obtain some Dppa4-/- animals these were very rare. Indeed we were only able to obtain 3 -/- males and all the information on the matings they were used in is shown in Figure 4C,D.

14. Lines 205-212: The fertility of Dppa4-/- females should be discussed.
This data was included in Figure 4F but not discussed in the text. We have now updated the appropriate section to include a comment on female fertility (see lines 206-208) and included the data on individual litter size from Dppa4-/- females mated with WT males in figure 4C.

15. Fig. S2B: The font size of gene labels should be increased.
We have done this as the reviewer suggested.

16. Line 241: the authors mentioned “maternal DPPA4 is important for offspring survival, and the complete absence of both maternal and embryonic DPPA4 is not compatible with development.” Is DPPA4 correct? It appears that some Dppa4-/- adults were obtained, but no Dppa2-/- adults
We apologise for the confusion in this statement and have revised this paragraph to more accurately reflect our findings (see lines 239-247).

17. Careful copy-editing throughout would improve the manuscript. For example, there are multiple incomplete/errors in References.
We apologise for this and have gone through the references carefully and corrected any errors.

© 2021. Published by The Company of Biologists under the terms of the Creative Commons Attribution License (https://creativecommons.org/licenses/by/4.0/).

Reviewer 2 Advance Summary and Potential Significance to Field:
In this study, the authors performed a vey comprehensive experiment and proved that the maternal deletion of both Dppa2 and Dppa4 showed no obvious effect on the zygotic genome activation. The experiments were well designed and conducted, and the results are important for the field to re-consider the important regulators of ZGA in mammals. The manuscript would be acceptable after minor revision.

Reviewer 2 Comments for the Author:

Major concern:
In the previous study from the group has shown that Dppa2 and Dppa4 can activate Dux in 2-cell like ES cells. In the double CKO embryos, the authors only checked the expression of MERVL and if the expression of Dux is affected? The authors can present this result and give some interpretation in terms the difference from ES studies.
We thank the reviewer for their comments. In response to this concern we have included expression levels of Dux3f in Supplemental Figure 3B and also levels of 2C-like genes from ESC studies (see lines 184-186).

Second decision letter

MS ID#: DEVELOP/2021/200191

MS TITLE: Maternal Dppa2 and Dppa4 are dispensable for zygotic genome activation but important for offspring survival

AUTHORS: Oana Kubinyecz, Fatima Santos, Deborah Drage, Wolf Reik, and Melanie A Eckersley-Maslin
ARTICLE TYPE: Research Article

I am happy to tell you that your manuscript has been accepted for publication in Development, pending our standard ethics checks.

Reviewer 2

Advance summary and potential significance to field

The authors have performed additional experiments and successfully addressed all concerns raised by this reviewer.

Comments for the author

No.

© 2021. Published by The Company of Biologists under the terms of the Creative Commons Attribution License (https://creativecommons.org/licenses/by/4.0/).
